# Predictive model for severe thrombocytopenia after transfemoral transcatheter aortic valve replacement

**DOI:** 10.3389/fcvm.2023.1213248

**Published:** 2023-08-09

**Authors:** Shaoman Li, Yafeng Wu, Jinju Wang, Liping She, Xuemei Zheng

**Affiliations:** Department of Cardiology, Nanjing First Hospital, Nanjing Medical University, Nanjing, China

**Keywords:** transcatheter aortic valve replacement, platelet count, thrombocytopenia, predictive model, aortic regurgitation

## Abstract

The aim of this study was to develop a predictive model for severe thrombocytopenia after transfemoral transcatheter aortic valve replacement (TAVR). A total of 155 patients treated with TAVR at our center were retrospectively enrolled in this study. The incidence of severe thrombocytopenia after TAVR was 25.16%, and most patients suffered from severe thrombocytopenia on 4 days after procedure. Multivariate regression analysis showed that weight <60 kg, New York Heart Association Functional Classification (NYHAFC IV), major vascular complications, and lower first post-procedural platelet count were independent risk factors for severe thrombocytopenia after TAVR. The c-statistic for the area under the curve was 0.758, the sensitivity was 0.744, the specificity was 0.784, and the negative predictive value of the model was 91.38%. The overall predictive value was 76.77%. The predictive model developed from this cohort data could effectively identify patients at high risk of severe thrombocytopenia after TAVR, and might be applicable to patients with aortic regurgitation (AR) and severe thrombocytopenia with different definitions.

## 1. Introduction

A large body of evidence demonstrated that thrombocytopenia is inevitable after transcatheter aortic valve replacement (TAVR) (1–4), and that severe thrombocytopenia are significantly associated with worse clinical outcomes (1–3, 5–8). With the development of TAVR technology, TAVR is an established treatment for selected severe symptomatic aortic stenosis (AS), and its safety and efficacy in patients with aortic regurgitation (AR) has also been explored (9–11). Although platelet activation and systemic inflammatory response are possible explanations for the pathophysiology of thrombocytopenia after TAVR (2, 12, 13), however, the clinical characteristics of patients, the definition of severe thrombocytopenia and reported risk factors varied widely in previous studies (1–8, 14–17). At present, there is no clinical tool that can be used to assess the risk of severe thrombocytopenia after TAVR. Therefore, we attempted to further explore the risk factors of severe thrombocytopenia after transfemoral TAVR in patients with AS or AR, and develop a prediction model to predict the probability of severe thrombocytopenia after TAVR, so as to provide reference for clinical decision-making and prevention.

## 2. Materials and methods

### 2.1. Study design and data collection

Between January 2019 and 2023 January, 169 patients with severe aortic valve disease, including severe AS and AR, were treated with transfemoral TAVR at our center. Patients with periprocedural death and baseline platelet count <50 × 10^9^/L were excluded, and finally a total of 155 patients participated in the development of the predictive model. All patients received general anesthesia and unfractionated heparin to maintain a minimum active clotting time of more than 250 s. The type and size of the implanted valve is determined by the cardiologist. Femoral artery access and closure was performed with the PROGLIDE suture-mediated closure system (Abbott Vascular Inc.,Santa Clara, Ca, USA). The procedural time was recorded accoring to “Skin to skin” (time 0 was the opening of the operative pathway and the end time was the closing of the operative pathway). Preprocedural and postprocedural antiplatelet regimens are at the discretion of the cardiologist, except for those requiring chronic oral anticoagulation.

### 2.2. Event and thrombocytopenia criteria

Periprocedural endpoints such as major vascular complications and severe bleeding (Type II/III) were classified according to the definition of Valve Academic Research Consortium 3 (VARC-3)[ (18)]. The lowest recorded platelet count during hospitalization was the nadir platelet count. Formula for decreased platelet count (DPC): [%DPC = 100 × (baseline platelet count—nadir platelet count)/baseline platelet count]. We divided the study population into severe thrombocytopenia (STP) and non-severe thrombocytopenia (NSTP). Severe thrombocytopenia (STP) was defined as DPC > 50% and platelet count <100 × 10^9^/L based on previous studies (7, 19, 20).

### 2.3. Statistical analysis

Continuous variables were tested for normality using the Shapiro-Wilk test, and categorical variables were compared using chi-square statistics or Fisher's exact test. Data that did not satisfy the normal distribution were tested using the Mann-Whitney *U* test. The parameters with significant univariate analysis (*p* < 0.05) and the variables of interest were subjected to multivariate analysis using two-way-stepwise regression method before variable adjustment. After variable adjustment, the regression coefficient, OR value and confidence interval were calculated by Binary logistic regression. According to the predictive value of multivariate analysis, with STP as the endpoint, the receiver-operator characteristic curve (ROC) was generated, and the ROC was further evaluated by c-statistics. Sensitivity, specificity, negative predictive value, and positive predictive value were calculated by specific cut-off values, using Youden's index generated from the ROC based on STP prediction probabilities. All analyses were considered significant with a 2-tail *p* < 0.05. *The SPSSAU project(2021). SPSSAU(Version 21.0)[Online Application Software]. Retrieved from* https://www.spssau.com. were used to perform all statistical evaluations.

## 3. Results

### 3.1. The clinical characteristics

The clinical and procedural characteristics of the 155 participants are presented in Table 1. 96.8% of patients experienced varying decreased degrees of platelet count after TAVR. The total platelet count decreased by 40.2% ± 18.8% and the mean nadir platelet count was 100.26 ± 37.01 × 10^9^/L. The platelet count decreased by 33% ± 15% and the nadir platelet count was 112.67 ± 32.28 × 10^9^/L in NSTP group, while the platelet count decreased by 62% ± 9% and the nadir platelet count was 63.33 ± 22.98 × 10^9^/L in STP group (*p* < 0.01). STP occurred in 39 patients (25.16%), mainly on the fourth postprocedural day. The body weight of patients in STP group was significantly lower than that in NSTP group (59.41 ± 10.08 vs. 64.82 ± 9.82, *p* < 0.01). There was significant difference in New York Heart Association Functional Classification (NYHAFC) between the two groups (*p* < 0.01). The first postprocedural platelet count in STP group was significantly lower than that in NSTP group (116.92 ± 36.58 vs. 147.69 ± 38.43 × 10^9^/L, *p* < 0.001). The incidence of severe bleeding, major vascular complications and blood transfusion in STP group was higher than that in NSTP group (28.21% vs. 8.62%, *p* < 0.01; 17.95% vs. 2.59%, *p* < 0.001; 43.59% vs. 16.38%, *p* < 0.001). Clinical and procedural characteristics were almost similar between patients with AS and those with AR (Supplementary Table S1). There was no significant difference in the nadir platelet count (102.98 ± 35.15 vs. 99.18 ± 37.82  × 10^9^/L, *p* = 0.566) and the incidence of STP between two groups (22.73% vs. 26.12%, *p* = 0.660). However, patients with AS were more prone to severe bleeding (8.62% vs. 28.21%, *p* = 0.039).

**Table 1:** Baseline and procedural characteristics.

	NSTP (*n* = 116)	STP (*n* = 39)	*p*-value
Baseline clinical characteristics			
Age, years	71.91 ± 8.78	73.87 ± 6.84	0.205
Male (%)	54.31	46.15	0.381
BMI, kg/m^2^	23.99 ± 3.13	23.03 ± 3.05	0.100
Weight, kg	64.82 ± 9.82	59.41 ± 10.08	**<0.01**
Hypertension (%)	60.34	66.67	0.485
Diabetes mellitus (%)	17.24	23.08	0.422
Previous CABG (%)	0.86	0	0.564
Previous PCI (%)	14.66	12.82	0.778
Previous CVA (%)	15.52	23.08	0.285
Previous PVD (%)	4.31	5.13	0.833
Previous COPD (%)	5.17	12.82	0.109
Previous CKD (%)	12.07	2.56	0.083
Coronary artery disease (%)	39.66	28.21	0.202
Dyslipidemia (%)^a^	70.69	74.36	0.350
Atrial fibrillation(%)	24.14	25.64	0.851
NYHAFC (%)			**<0.01**
I	0.86	0	
II	38.79	20.51	
III	53.45	56.41	
IV	6.9	23.08	
DAPT (%)	40.52	38.46	0.822
LVEF,%	56.72 ± 9.28	57.49 ± 7.80	0.645
AR (%)	29.31	25.64	0.663
AS (%)	70.69	74.36	
Laboratory parameters			
Baseline hemoglobin, g/L	122.23 ± 16.52	123.26 ± 18.66	0.747
First post-procedural hemoglobin, g/L	109.81 ± 18.51	103.51 ± 17.13	0.063
Baseline platelet count, × 10^9^/L	171.56 ± 48.23	167.56 ± 44.62	0.649
First post-procedural platelet count, ×10^9^/L	147.69 ± 38.43	116.92 ± 36.58	**<0.001**
Creatinine, µmol/L	75.5 [63.0, 94.0]	72.0 [59.6, 87.0]	0.208
NT-proBNP > 1,800 pg/ml	39.66	41.03	0.88
Procedural characteristics			
Type II/III bleeding (%)	8.62	28.21	**<0.01**
Major vascular complications (%)	2.59	17.95	**<0.01**
Blood transfusion (%)	16.38	43.59	**<0.001**
Contrast volume (ml)	245.41 ± 38.17	245.64 ± 26.04	0.971
Procedural time, min	234.16 ± 58.52	251.54 ± 55.32	0.106
Balloon-expandable valves (%)	6.90	7.69	0.867
Self-expanding valves (%)	93.10	92.31	
Transcatheter aortic valves			
SAPIEN 3™ (%)	6.9	7.69	0.867
VitaFlow® (%)	81.03	74.36	0.373
TaurusElite® (%)	11.21	17.95	0.277
VenusA-Pro® (%)	0.86	0	0.561

Values are expressed as mean ± SD, *n* (%) or median (25th percentile, 75th percentile).

AR, Aortic valve regurgitation; AS, Aortic valve Stenosis; BMI, body mass index; CABG, coronary artery bypass graft; PCI, percutaneous coronary intervention; CVA, cerebrovascular accident; PVD, peripheral vascular disease; COPD, chronic obstructive pulmonary disease; CKD, chronic kidney disease; LVEF, left ventricular ejection fraction; NYHAFC, New York Heart Association Functional Classification; DAPT, dual antiplatelet therapy; NT-proBNP, N-Terminal Pro-Brain Natriuretic Peptide.

Bold indicates the statistically significant differences.

^a^Low-density lipoprotein >3.10 mmol/l.

### 3.2. Variable analysis

The results of multivariate analysis before and after variable adjustment are shown in Table 2. NYHAFC (*p* < 0.01), first post-procedural platelet count (*p* < 0.001), and major vascular complications (*p* < 0.01) were significantly associated with STP before adjustment, with the highest correlation being for major vascular complications (coefficient: 0.431, *p* < 0.01). Multivariate analysis after variable transformation of body weight, NYHAFC, and first post-procedural platelet count showed that body weight <60 kg (*p* = 0.02), NYHAFC IV (*p* < 0.01), low first post-procedural platelet count (*p* < 0.01), and major vascular complications (*p* < 0.01) were independent risk factors for STP after TAVR, accounting for 28.4% of the change in STP. Binary logistic regression showed weight <60 kg was 2.669 times higher of STP after TAVR compared with weight ≥60 kg (OR:2.669; 95% CI: 1.134–6.285; *p* = 0.025). Patients with NYHAFC IV was associated with 5.380 times higher risk of STP after TAVR than NYHAFC I-III (OR:5.380; 95% CI: 1.732–16.716; *p* = 0.004). Moreover, patients with major vascular complications (OR: 6.887; 95%CI: 1.496–31.699; *p* = 0.013) had a nearly 7 times increased risk of STP during hospitalization.

**Table 2:** Multivariate analyses of the predictors of STP.

Variables	Unadjusted		Variables	Adjusted			
	Coefficient	*p*-value		Coefficient	*p*-value	OR	95% CI
Weight, kg	–	–	Weight, kg	0.982	0.025	2.669	1.134–6.285
			<60 kg				
			≥60 kg				
NYHAFC I—IV	0.126	<0.01	NYHAFC IV	1.683	0.004	5.38	1.732–16.716
First post-procedural platelet count,×10°/L	−0.003	<0.001	First post-procedural platelet count, ×10°/L	0.817	0.006	0.442	0.246–0.794
			≥150				
			≥100, <150				
			<100				
Major vascular complications (%)	0.431	<0.01	Major vascular complications (%)	1.930	0.013	6.887	1.496–31.699

CI, confidence interval; OR, odds ratio; NYHAFC, New York Heart Association Functional Classification.

### 3.3. Model development and performance

Due to a previously reported incidence of STP of 26.9%–56.5% (3, 14, 17), we use 30% probability and 10 events per parameter (EPP) rule to develop the predictive model with four parameters included. The criteria and results of the test are shown in Table 3, including STP, platelet count <100 × 10^9^/L, platelet count <50 × 10^9^/L, DPC > 30%, and DPC > 50%. The model had better discrimination for STP during hospitalization (AUC = 0.758; 95% CI: 0.657–0.859, *p* < 0.001), with a sensitivity of 0.744, a specificity of 0.784, a negative predictive value of 91.38%, a positive predictive value of 33.33%, and the overall probability of 76.77%. There was better discrimination for platelet count <100 × 10^9^/L (AUC = 0.732; 95% CI: 0.651–0.813, *p* < 0.001) and platelet count <50 × 10^9^/L (AUC = 0.862; 95% CI: 0.754–0.970, *p* < 0.001). However, the discrimination ability of DPC > 30% (AUC = 0.649; 95% CI: 0.560–0.738, *p* < 0.01) and DPC > 50% (AUC = 0.676; 95% CI: 0.569–0.783, *p* < 0.01) was not significant. Finally, based on the regression coefficients of multivariate analysis, we created a scoring chart to provide reference for clinical decision-making and prevention, as seen in Table 4. The estimation of scores and prediction probabilities is based on the Framingham Study Risk Score (21).

**Table 3:** C-statistics to STP with different definitions.

Definition	Cases (%)	AUC	Std. Error	*p*-value	95% CI
STP	39 (25.16)	0.758	0.051	<0.001	0.657–0.859
Platelet count <100 × 10^9^/L	84 (54.19)	0.732	0.041	<0.001	0.651–0.813
Platelet count <50 × 10^9^/L	12 (7.74)	0.862	0.055	<0.001	0.754–0.970
DPC > 30%	117 (75.48)	0.649	0.045	<0.01	0.560–0.738
DPC > 50%	46 (29.68)	0.676	0.055	<0.01	0.569–0.783

**Table 4:** Scoring chart of STP during hospitalization after TAVR (PREDICT—STP).

Items	Points	Total score	Probability of prediction
Weight, kg			
<60 kg	2	2	14.74%–16.94%
≥60 kg	0	4	28.12%–31.57%
NYHAFC IV		5	34.47%
Yes	4		
No	0	6	48.19%–52.31%
First post-procedural		7	54.35%–58.41%
platelet count, ×10°/L		8	67.79%–71.29%
≥150	0	9	72.93%–76.06%
≥100, <150	2		
<100	4	10	84.89%
Major vascular complications		11	87.79%–88.31%
Yes	5	13	94.47%
No	0	15	97.48%

NYHAFC, New York Heart Association Functional Classification.

## 4. Discussion

The findings of this study were as follows: (1) The incidence of STP after TAVR was 25.16%, and the overall platelet count decreased by 40.2% ± 18.8%; (2) There was no significant difference in the incidence of STP after TAVR between AR and AS patients; (3) Independent risk factors for STP after TAVR were weight <60 kg, NYHAFC IV, first post-procedural platelet count and major vascular complications; (4) The combination of these risk factors can effectively predict STP after TAVR, and has certain predictive value for thrombocytopenia under different criteria.

It still remains controversial that patients with lower weight are associated with thrombocytopenia after TAVR. Eight studies have evaluated the effect of BMI on thrombocytopenia after TAVR (1, 3, 4, 6–8, 16, 19), and only two have shown a significant association between BMI and thrombocytopenia. Although almost all studies used BMI as a measurement of body weight and our study showed no association between BMI and STP, there was a significant correlation when the body weight was less than 60 kg. This leads us to wonder whether STP after TAVR is really independent of body weight. The main explanations for STP in low-weight patients after TAVR are platelet loss and hemodilution. As our study could not fully record the blood loss during the procedural, and STP is unlikely to be related to platelet loss based on the results of hemoglobin reduction before and after procedure, which is consistent with the report of Yamada et al. (17). Thus, the most likely explanation is hemodilution, which explains why our findings are not related to BMI, but are significantly related to the body weight. Body weight is a better indicator of a person's volume and blood volume than BMI. Patients with lower weight have smaller volumes, and large amounts of perioperative fluids have a more significant effect on hemodilution. Although we cannot exclude the differences in weight and infusion management after TAVR between Asian and European populations, in any case, patients with lower weight are more likely to have adverse outcomes after TAVR (22).

The present study found an association between vascular complications and STP after TAVR, which is consistent with previous studies, and relevant studies described its possible mechanism as platelet activation and hemodilution (6, 14). In addition, the occurrence of STP after TAVR may be the result of rapid platelet depletion in adverse events, including vascular complications and bleeding, and can be considered as a marker of systemic inflammatory response after TAVR (14, 23).

To our knowledge, this is the first study to report an association between first post-procedural platelet count and in-hospital STP. The first post-procedural platelet count after TAVR may be the result of physiological stress reaction under the combined action of different events such as procedural events, such as vascular complications, bleeding, blood transfusion, etc. This is likely to be a very sensitive marker reflecting the immediate clinical status of patients after TAVR. Therefore, it is logical that patients with lower first post-procedural platelet count after TAVR are more likely to develop severe thrombocytopenia.

Previous studies have reported LVEF as an independent risk factor for STP (5). Although we tried to use LVEF and NT-proBNP to more objectively reflect the cardiac function and evaluate the effect of deterioration of cardiac function on STP, the results showed that NYHAFC IV had a more positive effect. We have not found relevant studies describing the mechanism of STP after TAVR in patients with heart failure, and recent studies have shown a strong correlation between left ventricular end-diastolic pressure increase and platelet count decrease in patients with heart failure, suggesting that pressure overload of the lung may interfere with the function of the lung as a site of platelet biosynthesis (24).

Of particular note, this is the first report to compare STP after TAVR in patients with AR and AS, and this is a good response to the controversy about the role of endothelial injury and shear stress in STP after TAVR (1, 5, 16, 17). In theory, endothelial injury and shear stress after valve release should be more pronounced in patients with AS, but the final results showed no correlation between AS and AR. Due to the small sample size and limited statistical ability of our study, the controversy surrounding the explanation of endothelial damage and shear stress still requires future research to verify.

The model did not perform as expected in DPC > 30% and DPC > 50%. Considering that the average platelet decrease in our study was 40.2% ± 18.8%, so we focused on DPC > 50%, which is a more clinically meaningful index. Although DPC > 50% may reflect a decrease in platelet count due to some factors in this group of patients, it cannot be ruled out that platelet count may be normal in a significant proportion of these patients. Therefore, DPC > 50% as an outcome index cannot explain the confusion in clinical diagnosis and treatment. Of course, the best definition of severe thrombocytopenia after TAVR and its associated prognosis remain to be determined. Although our goal is to maximize the clinical value of our findings and enable patients to recover safely and quickly, the statistical power of the study may be insufficient due to sample size and population limitations, and more data are needed to validate and optimize the model to help more patients.

## 5. Conclusions

Severe thrombocytopenia occurring at high rates after TAVR, is consistently associated with poor clinical outcomes. This study demonstrated that weight <60 kg, NYHAFC IV, major vacular complications, and lower first post-procedural platelet count were independent risk factors for severe thrombocytopenia after TAVR. Further clinical trials are warranted to validate these findings.

## Data Availability

The original contributions presented in the study are included in the article/**Supplementary Material**, further inquiries can be directed to the corresponding authors.
