# Supplementary material for: Predictive model for severe thrombocytopenia after transfemoral transcatheter aortic valve replacement
Source: Front Cardiovasc Med. 2023 Aug 9;10:1213248. doi: 10.3389/fcvm.2023.1213248 (PMC10449450; doi:10.3389/fcvm.2023.1213248)
Supplement: Supplementary file 1 [file Table1.docx]

| **Supplemental Table 1. Baseline and Procedural Characteristics AR vs AS** | | | |
| --- | --- | --- | --- |
|  | **AR (n=44)** | **AS (n=111)** | **p value** |
| **Clinical characteristics** |  |  |  |
| Age, years | 72.16±8.23 | 72.50±8.45 | 0.822 |
| Male (%) | 59.09 | 49.55 | 0.284 |
| BMI, kg/m2 | 23.10±3.15 | 24.00±3.09 | 0.107 |
| Weight, kg | 61.73±10.57 | 64.14±9.92 | 0.181 |
| Hypertension (%) | 70.45 | 58.56 | 0.169 |
| Diabetes mellitus (%) | 15.91 | 19.82 | 0.574 |
| Previous CABG (%) | 0 | 0.9 | 0.528 |
| Previous PCI (%) | 13.64 | 14.41 | 0.900 |
| Previous CVA (%) | 20.45 | 16.22 | 0.530 |
| Previous PVD (%) | 6.82 | 3.6 | 0.385 |
| Previous COPD (%) | 6.82 | 7.21 | 0.932 |
| Previous CKD (%) | 11.36 | 9.01 | 0.655 |
| Coronary artery disease(%) | 34.09 | 37.84 | 0.663 |
| Dyslipidemia (%)† | 11.36 | 20.72 | 0.172 |
| Atrial fibrillation(%) | 27.27 | 23.42 | 0.615 |
| NYHAFC (%) |  |  | 0.939 |
| I | 0 | 0.9 |  |
| II | 34.09 | 34.23 |  |
| III | 54.55 | 54.05 |  |
| IV | 11.36 | 10.81 |  |
| DAPT (%) | 31.82 | 43.24 | 0.190 |
| LVEF,% | 55.98±8.36 | 57.29±9.13 | 0.411 |
| Baseline Hemoglobin, g/L | 117.84±18.35 | 124.33±16.20 | **0.032** |
| First post-procedural Hemoglobin, g/L | 108.84±19.08 | 107.98±18.10 | 0.793 |
| Baseline platelet count, ×10°/L | 171.02±43.48 | 170.37±48.84 | 0.938 |
| First post-procedural platelet count, ×10°/L | 146.98±43.73 | 137.16±38.50 | 0.171 |
| Creatinine, umole/L | 75.0[65.7,87.9] | 75.0[62.0,94.0] | 0.790 |
| NT-proBNP>1800pg/ml | 22.73 | 46.85 | **<0.01** |
| Nadir platelet count, ×10°/L | 102.98±35.15 | 99.18±37.82 | 0.566 |
| **Procedural characteristics** |  |  |  |
| Type II/III bleeding (%) | 4.55 | 17.12 | **0.039** |
| Major vascular complications (%) | 2.27 | 8.11 | 0.182 |
| Blood transfusion (%) | 15.91 | 26.13 |  |
| Contrast volume (ml) | 253.41±46.85 | 242.32±29.39 | 0.079 |
| Procedural time (min) | 234.14±55.33 | 240.27±59.24 | 0.555 |
| STP (%) | 22.73 | 26.13 | 0.660 |
| Values are expressed as mean ± SD, n (%) or median (25th percentile, 75th percentile).  AR = Aortic valve regurgitation; AS = Aortic valve Stenosis; BMI = body mass index; CABG = coronary artery bypass graft;  PCI = percutaneous coronary intervention; CVA = cerebrovascular accident; PVD = peripheral vascular disease;  COPD = chronic obstructive pulmonary disease; CKD = chronic kidney disease; LVEF = left ventricular ejection fraction;  NYHAFC = New York Heart Association Functional Classification; DAPT = dual antiplatelet therapy;  NT-proBNP = N-Terminal Pro-Brain Natriuretic Peptide. | | | |
